# A photo-triggering double cross-linked adhesive, antibacterial, and biocompatible hydrogel for wound healing

**DOI:** 10.1016/j.isci.2022.104619

**Published:** 2022-06-16

**Authors:** Honghua Hu, Xinrang Zhai, Wenyue Li, Shunxian Ji, Wei Dong, Weiyu Chen, Wei Wei, Zhongfa Lu

**Affiliations:** 1 International Institutes of Medicine, the Fourth Affiliated Hospital, Zhejiang University School of Medicine, Yiwu, Zhejiang 322000, China; 2 Key Laboratory of Tissue Engineering and Regenerative Medicine of Zhejiang Province, Zhejiang University School of Medicine, Hangzhou, Zhejiang 310000, China; 3 Zhejiang University-University of Edinburgh Institute, Zhejiang University, Hangzhou, Zhejiang 310000, China; 4 School of Chemistry and Chemical Engineering, Nanjing University of Science and Technology, Nanjing, Jiangsu 210094, China; 5 Department of Dermatology, the Second Affiliated Hospital, Zhejiang University School of Medicine, Hangzhou, Zhejiang 310000, China

**Keywords:** Biotechnology, Applied sciences, Bioengineering, Materials science, Biomaterials

## Abstract

Full-thickness wounds, lacking the epidermis and entire dermis and extending into subcutaneous fat, represent a common treatment challenge. Due to the loss of adnexal structures as a source of keratinocytes, full-thickness wounds healing can only be achieved by re-epithelialization from the wound edge and contraction. Here, we developed a hydrogel composed of chitosan methacrylate (CSMA) and *o*-nitrosobenzaldehyde-modified gelatin (GelNB) for promoting full-thickness wound healing. The CSMA/GelNB (CM/GN) hydrogels exhibited superior mechanical and adhesive properties than that of pure CSMA hydrogel. *In vivo* experiments confirmed that CM/GN could promote wound healing by generating more hair follicles and mutual blood vessels, high fibroblasts density, and thicker granulation tissue thickness. In addition, reduced secretions of tumor necrosis factor-α (TNF-α) and enhanced secretions of vascular endothelial growth factor (VEGF) could be observed in regenerated tissues after CM/GN treatment. These results suggested that CM/GN hydrogels could be promising candidates to promote wound healing.

## Highlights

• The CM/GN hydrogels exhibited tissue adhesive properties
• CM/GN hydrogel facilitated the proliferation of bone marrow stem cells
• CM/GN hydrogel efficiently promote full-thickness wound healing
• More hair follicles and mutual blood vessels were generated during wound healing

## Graphical abstract

**Figure:**
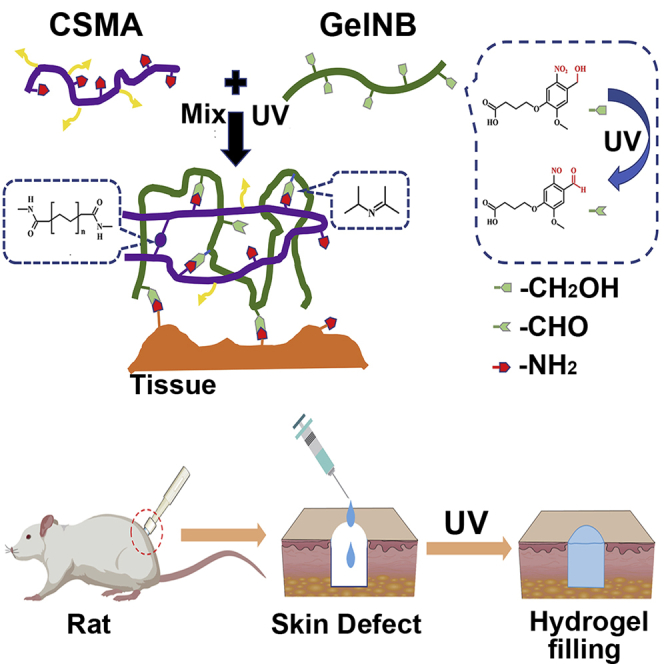

## Introduction

Skin damage is one type of the most common physical injuries (Xu et al., 2015; Zhao et al., 2017) and could be caused by various factors, such as abrasion, empyrosis, and clinical operation. At present, the wound dressing is recognized as an effective treatment option in clinical practice. Alcohol or iodize for sterilization and cotton gauze for fixation is applied widely in skin treatment (Yang et al., 2021). However, cotton gauze fixation is limited at certain areas, like axilla and ankle, because it would be easy to fall off in daily activities. Tedious replacement is required due to non-antibacterial property itself. Moreover, the wound healing process is complex so that efficient products need to address issues such as bacterial infection, biofilm formation, impaired angiogenesis, and prolonged inflammation with wounds simultaneously (Atashgah et al., 2021; Matoori et al., 2021). Therefore, new biomaterials with multi-functions for skin repair are highly desired for clinical applications.

Hydrogels have attracted increasing attention in recent years for their unique properties such as high-water content, viscoelastic, and biocompatibility (Gong et al., 2013; Guo et al., 2013; Wei et al., 2021). The porous structure and suitable swelling ratio support various biomedical applications such as biosensing (Choi et al., 2020; Mao et al., 2021), drug delivery (Zhao et al., 2021), tissue engineering (Ghorbani et al., 2020), and microenvironment regulation (Fan et al., 2014). Moreover, numerous surface groups ensure the combination of hydrogels with functional materials to enhance mechanical property and treatment effect (Kwak et al., 2009; Van Nieuwenhove et al., 2016; Ahmad et al., 2017). Various materials, such as silk fibroin (Mitropoulos et al., 2015), gelatin (Huang et al., 2019), chitosan (Osi et al., 2021), and hyaluronic acid (Koivusalo et al., 2019), have been utilized in practice. Among these commonly used precursors, gelatin has been considered a most promising candidate for skin treatment due to its excellent biocompatibility, abundant surface groups, and high solubility (Yue et al., 2015). Numerous gelatin-based composites have been explored through combination with functional materials, like methacrylate modification (GelMA) (Yin et al., 2018), stearic acid modification (Karnnet et al., 2005), carbohydrazide modification (Heo et al., 2020), epigallocatechin gallate modification (Honda et al., 2018), and heparin modification (Niu et al., 2014). Previous works have obtained diverse gelatin-based hydrogels with strong mechanical strength, antioxidative activity, and outstanding emulsifying properties (Rose et al., 2014; Suderman et al., 2018).

For wound dressing, the adhesive property is another vital point worth to be considered (Balakrishnan et al., 2014). For example, Liu et, al. fabricated an adhesive hydrogel for diabetic foot wound dressing by combining polyacrylamide, gelatin with ε-polylysine (Liu et al., 2021). Blacklow et, al. reported an active adhesive dressing assembled with functional components to accelerate wound closure (Blacklow et al., 2019). Recently, N-(2-aminoethyl)−4-(4-(hydroxymethyl)−2-methoxy-5-nitrosophenoxy) butanamide (NB) has been demonstrated as a tissue adhesive functional molecule because benzyl alcohol structure of NB would be transferred into benzaldehyde structure under UV irradiation, which is contributed to interact with amino groups of tissue (Hong et al., 2019). Therefore, NB could be a promising candidate to modify the gelatin to form a biomacromolecule with tissue adhesive properties.

Besides, antibacterial properties are also favorable for wound dressing materials (Sun et al., 2021). Chitosan (CS), a polysaccharide, has been proved to have remarkable antibacterial capacity and reaction activity with negatively charged compounds owing to the polymeric cationic characteristics (Douglas et al., 2006; Attasgah et al., 2022). However, CS can only be dissolved in acid solution, but rarely in neutral solution and interstitial fluid (Geng et al., 2005), which hinders the application in skin treatment. The CS modified with methacrylate (MA) design is a pretty solution (Kolawole et al., 2018). The CS methacrylate (CSMA) avoids strict pH limitations and realizes favorable compatibility with medical hydrogels.

Herein, photo-triggering CSMA/GelNB (CM/GN) hydrogels were fabricated for wound dressing: (1) CSMA was used as photo-crosslinkable and antibacterial agent for *in situ* hydrogel formation and avoiding bacterial infection, (2) GelNB consists of gelatin and NB could provide cell adhesion and wet tissue adhesive properties simultaneously to promote tissue regeneration and integration. To our knowledge, this is the first time to fabricate a hydrogel adhesive based on CSMA and GelNB for wound healing. The prepared CSMA and GelNB were characterized by ^1^H-NMR, while CM/GN hydrogels were characterized by SEM and FTIR spectroscopy. Moreover, mechanical strength and adhesive property were evaluated. Antibacterial properties were tested based on *Escherichia coli (E. coli)*. *In vitro* study based on bone marrow stem cells (BMSCs) was also conducted. Finally, the wound healing properties of CM/GN hydrogel were studied *in vivo*. The therapeutic effects of CM/GN hydrogels were evaluated by histological and immunofluorescence (IF) staining.

## Results and discussion

**Figure 1:**
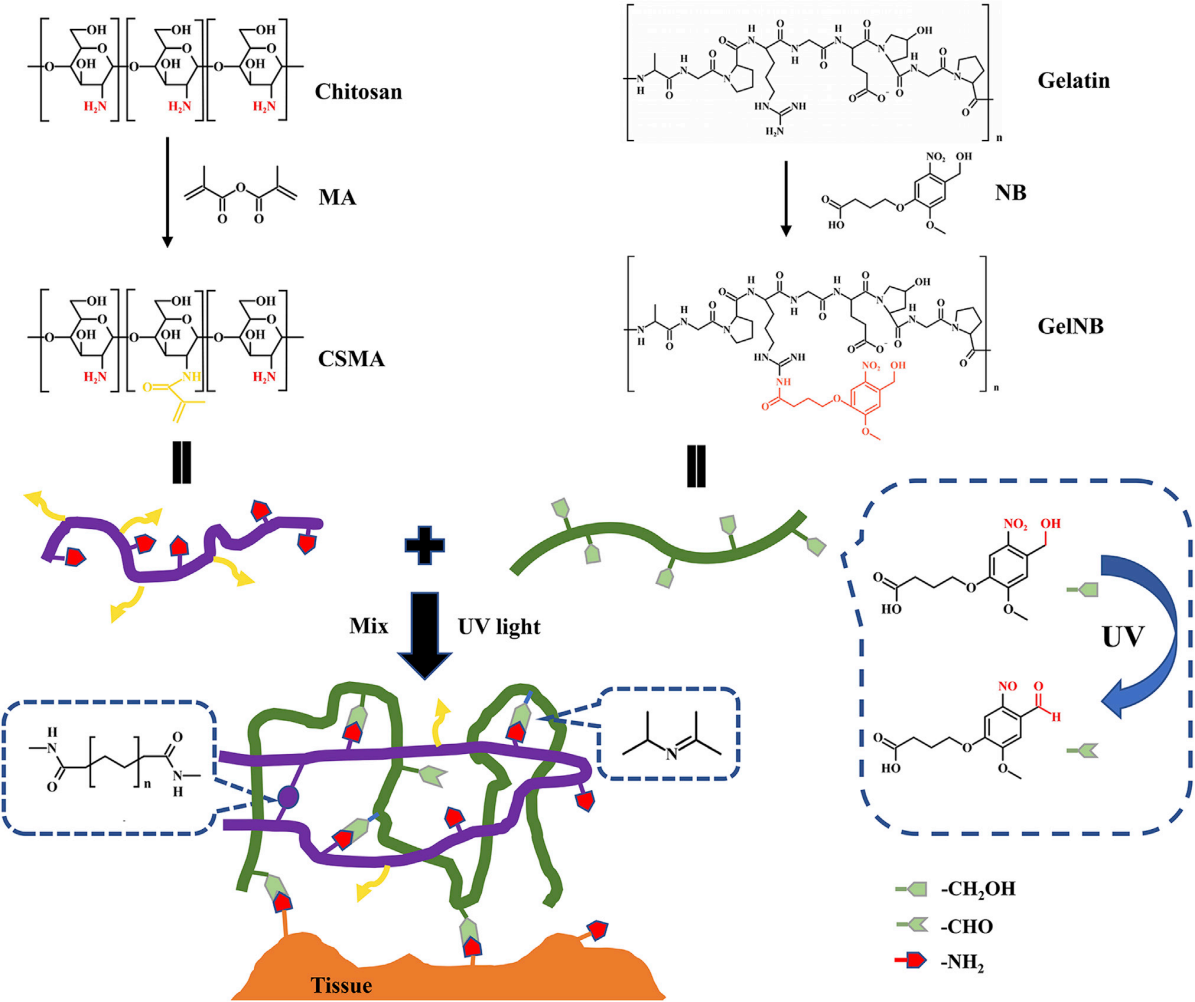
Schematic illustration of the fabrication of CM/GN hydrogels

To fabricate the photo-triggering double cross-linked adhesive, antibacterial, and biocompatible hydrogel, CSMA and GelNB were first synthesized. The chemical process of the hydrogel network formation is illustrated in Figure 1. For CSMA synthesis, the primary amino groups of chitosan were reacted with MA through amidation. To successfully synthesize GelNB, the molecule NB was designed with a carboxyl group to connect to the amino groups in the gelatin. The previously reported amino-terminal NB (Hong et al., 2019) could not be used to synthesize GelNB because there are both amino groups and carboxyl groups in the molecular structure of gelatin that could directly react during the activation of EDC/NHS. Therefore, in this study, the carboxyl terminal NB was first active by EDC/NHS and then grafted to the gelatin.

To form a double cross-linked multi-functional hydrogel, CSMA, GelNB, and photo-initiator LAP were mixed. After UV light irradiation, LAP was cleavaged into free radicals and initiated the polymerization of CSMA that formed the first network (Fairbanks et al., 2009). Simultaneously, the grafted NB was also triggered by the UV light and formed aldehyde groups that could bond the amino groups in CSMA through Schiff base reaction, forming the secondary network. Furthermore, the UV-triggered GelNB could simultaneously bind to the tissue surface by a similar mechanism (Yang et al., 2016). Hereby, the tissue adhesive and biocompatible hydrogel was fabricated based on natural macromolecule derivatives and photo-triggering cross-linking.

**Figure 2:**
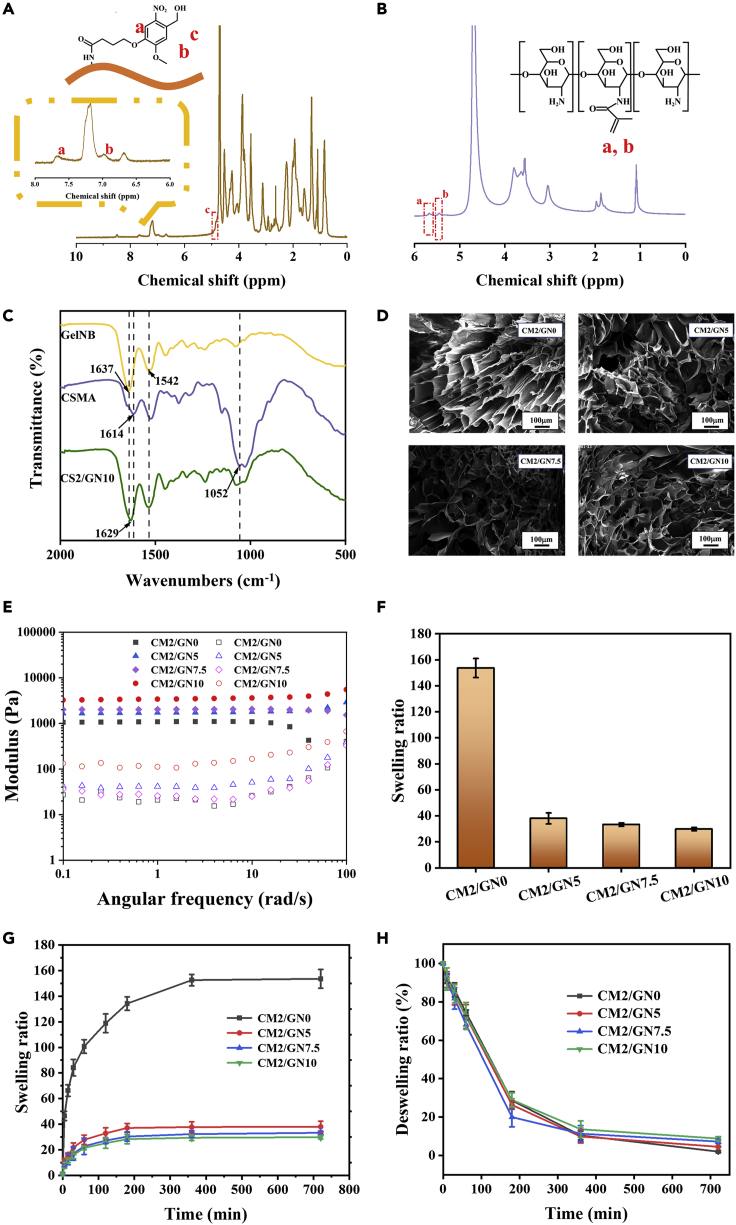
Characterizations of synthesized biomacromolecules and hydrogels ^1^H-NMR spectrum of (A) GelNB and (B) CSMA. (C) FT-IR spectra of GelNB, CSMA, and CM2/GN10 hydrogel. (D) SEM of CM/GN hydrogels. Scale bar: 100 μm. (E) Storage modulus (solid symbols) and loss modulus (open symbols) of the hydrogels in angular frequency range of 0.1–100 rad/s. (F) Equilibrium swelling ratios, (G) Swelling kinetics curves, and (H) Deswelling kinetics curves of the hydrogels. All data are presented as the mean ± SD.

The successful synthesis of GelNB and CSMA was proved by ^1^H-NMR. As shown in Figure 2A, the peaks of protons at 7.6 ppm (peak a), 7.0 ppm (peak b), and 4.8 ppm (peak c) are the characteristic peaks of NB, suggesting the achievement of NB modification onto the molecular chain of gelatin. Also, the peaks of protons at around 5.5 ppm (peak a and b) (Figure 2B) were observed in ^1^H-NMR spectrum of CSMA, demonstrating that vinyl groups were found on the molecular chains (Osi et al., 2021). The results exhibited the reliability of precursor used in subsequent experiments.

After the formation of photo-triggering hydrogels, the functional groups of the material were analyzed by FT-IR spectra (Figures 2C and S1). In the spectrum of GelNB, the peaks near 1637 cm^−1^ and 1542 cm^−1^ were ascribed to prominent amine of gelatin (Xavier et al., 2015). Also, CSMA spectrum exhibited the characteristic peaks at 1052 cm^−1^ (C-*O*-C bridge symmetric stretching) and 1614 cm^−1^ (C=O stretching, amide I band) (Osi et al., 2021). In the spectrum of CM/GN hydrogel samples, both characteristic peaks of CSMA (1052 cm^−1^ for C-*O*-C bridge symmetric stretching) and GelNB (1629 cm^−1^ for amino group of gelatins) were observed, indicating the material was successfully fabricated by CSMA and GelNB. The intact FTIR spectra can be found in Figure S1. The microstructure of prepared hydrogels was exhibited by SEM. As shown in Figure 2D, the pore channels existed in the series of hydrogels. The porous structure of the adhesive hydrogels ensured the routes of H_2_O molecules and therapeutic factors, indicating that the series of CM/GN hydrogels was potential in skin treatment (Nguyen and Alsberg, 2014). In addition, rheology test was performed to the hydrogel samples and the result was shown in Figure 2E. The storage modulus (G′) is exhibited as greater than loss modulus (G″) in all the samples, demonstrating the materials were gel state (Wei et al., 2015). Swelling properties and water content were also important factors for hydrogel materials. As displayed in Figures 2F and S2, CM2/GN0 showed an equilibrium swelling ratio up to 160 (water content was as high as 97.87%) while that of CM/GN hydrogels was less than 40. Swelling kinetics tests exhibited that these hydrogels were able to fast absorb water into their network in 6 h (Figure 2G). When put the equilibrium swelled hydrogels in the air, they showed similar deswelling kinetics curves (Figure 2H). The phenomenon that water content of prepared hydrogels decreased along with the increasing GelNB during fabrication process could be explained that stronger cross-linking contributed to high polymer density and small-sized pore, restricting water penetration (Qi et al., 2015).

**Figure 3:**
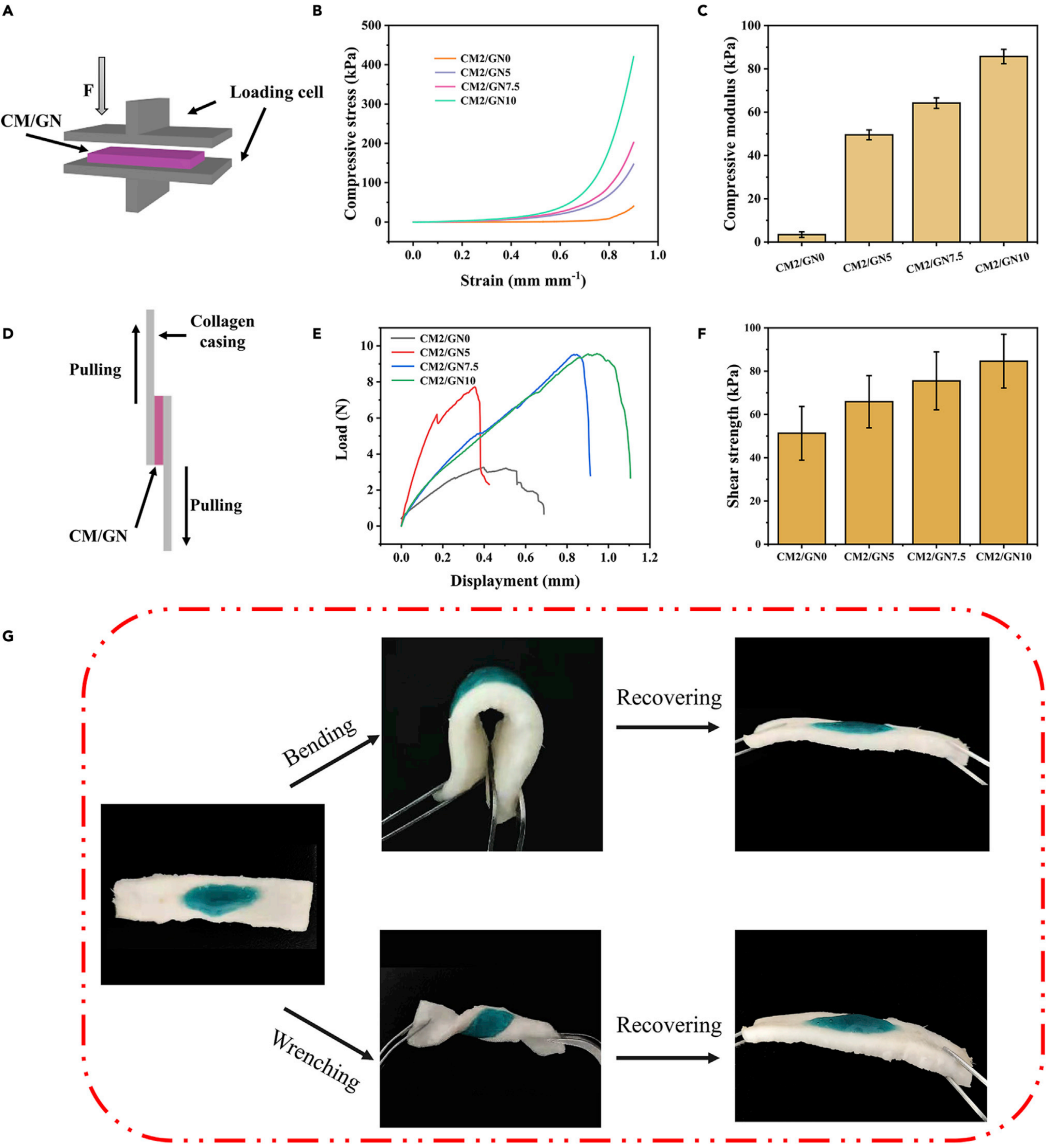
Mechanical properties and tissue adhesive properties of hydrogels (A) Schematic illustration of compressive test. (B) Stress/strain curves of CM/GN hydrogels from compressive test. (C) Compressive modulus of prepared hydrogels. (D) Schematic illustration of adhesive property test. (E) Curve of adhesive force between the hydrogels and substrates. (F) Adhesive strength of prepared hydrogels. (G) Photos of CM2/GN10 synthesized *in situ* on porcine skin, and recovered after bending and wrenching. All data are presented as the mean ± SD.

The mechanical properties of the hydrogel were evaluated by a coaxial compressive test as shown in Figure 3A. All the tested hydrogel samples, with different content of GelNB, were able to withstand up to 90% strain without fracture (Figure 3B). Nevertheless, the stress/strain curve indicated the modulus of the hydrogels could be influenced by the content of GelNB. After calculation, the compressive modulus of CM2/GN0, CM2/GN5, CM2/GN7.5, and CM2/GN10 was 3.40 kPa, 49.52 kPa, 64.18 kPa, and 85.72 kPa, respectively (Figure 3C). This could be explained as the solid content of hydrogel increased from CM2/GN0 to CM2/GN10. Besides, the cross-linking of GelNB network and the formation of double polymer networks also contribute to the improvement of mechanical strength.

To investigate the tissue adhesive properties, lap shear tests were conducted according to a modified ASTM standard (F2255-05) (Figure 3D). It was obvious that the more GelNB was added in precursor, the stronger adhesion CM/GN possessed (Figures 3E and 3F). It was explained that high GelNB content supported more aldehyde groups to react with CSMA amino groups, resulting in stronger cross-linking. Moreover, the more aldehyde groups could also lead to more imine connection to the collagen casing. These two reasons both contribute to the strong adhesion of CM/GN hydrogel to tissue surface (Yuk et al., 2016). Furthermore, CM2/GN10 was selected to test the tissue adhesive property to porcine skin *ex vivo* (Figure 3G). The CM2/GN10 pre-gel solution was applied to the porcine skin and *in situ* formed a hydrogel adhesive under a UV light. For better visualization, an edible pigment was added into the pre-gel solution. As shown in Figure 3G, the CM2/GN10 exhibited flexible mechanical property and strong adhesion to the porcine skin, and it could be recovered into its original status after bending and wrenching.

**Figure 4:**
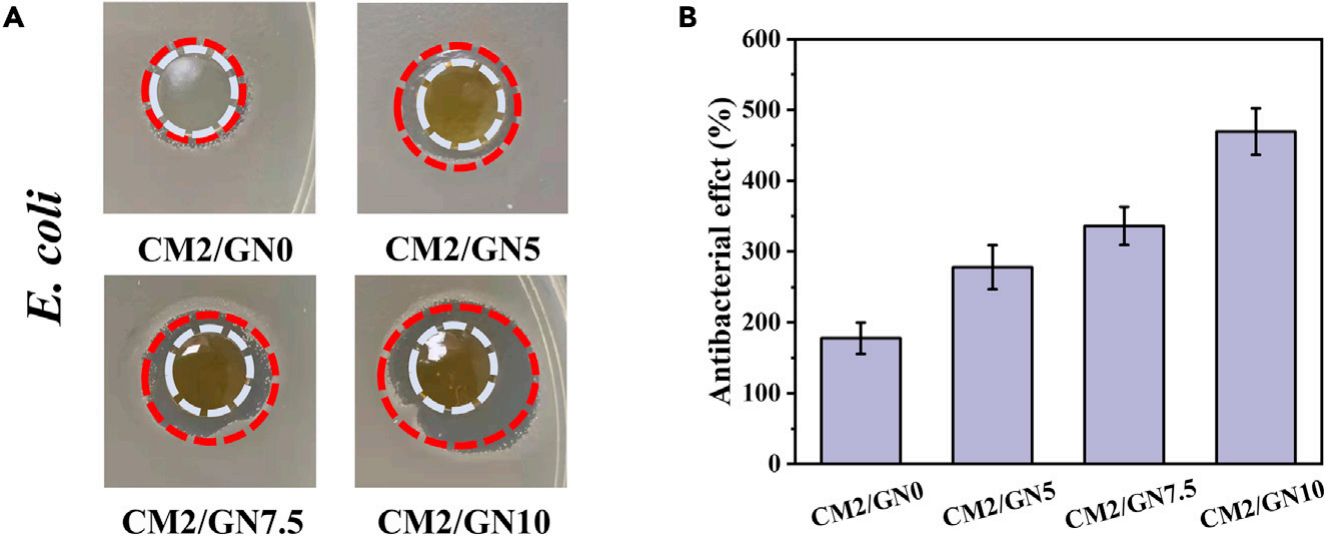
Antibacterial property of CM/GN hydrogels Photographs of the area that CM/GN inhibited *E. coli* proliferation (A), antibacterial activity against *E. coli* (B). All data are presented as the mean ± SD.

Inherent antibacterial property of wound dressing was a most attractive field for skin repair. The antibacterial effect of CM/GN hydrogels against *E. coli* was evaluated by the area *E. coli* proliferating. As displayed in Figures 4A and 4B, the area CM/GN hydrogels affected was 177.8% (CM2/GN0), 277.8% (CM2/GN5), 336.1% (CM2/GN7.5), and 469.4% (CM2/GN10), respectively. It was demonstrated that the synthesized wound dressing possessed excellent antibacterial capacity and the addition of GelNB was beneficial to inhibit *E. coli* proliferation. The nitric oxide and aldehyde groups formed by NB could contribute to the antibacterial property of this hydrogel system (Duan et al., 2021).

**Figure 5:**
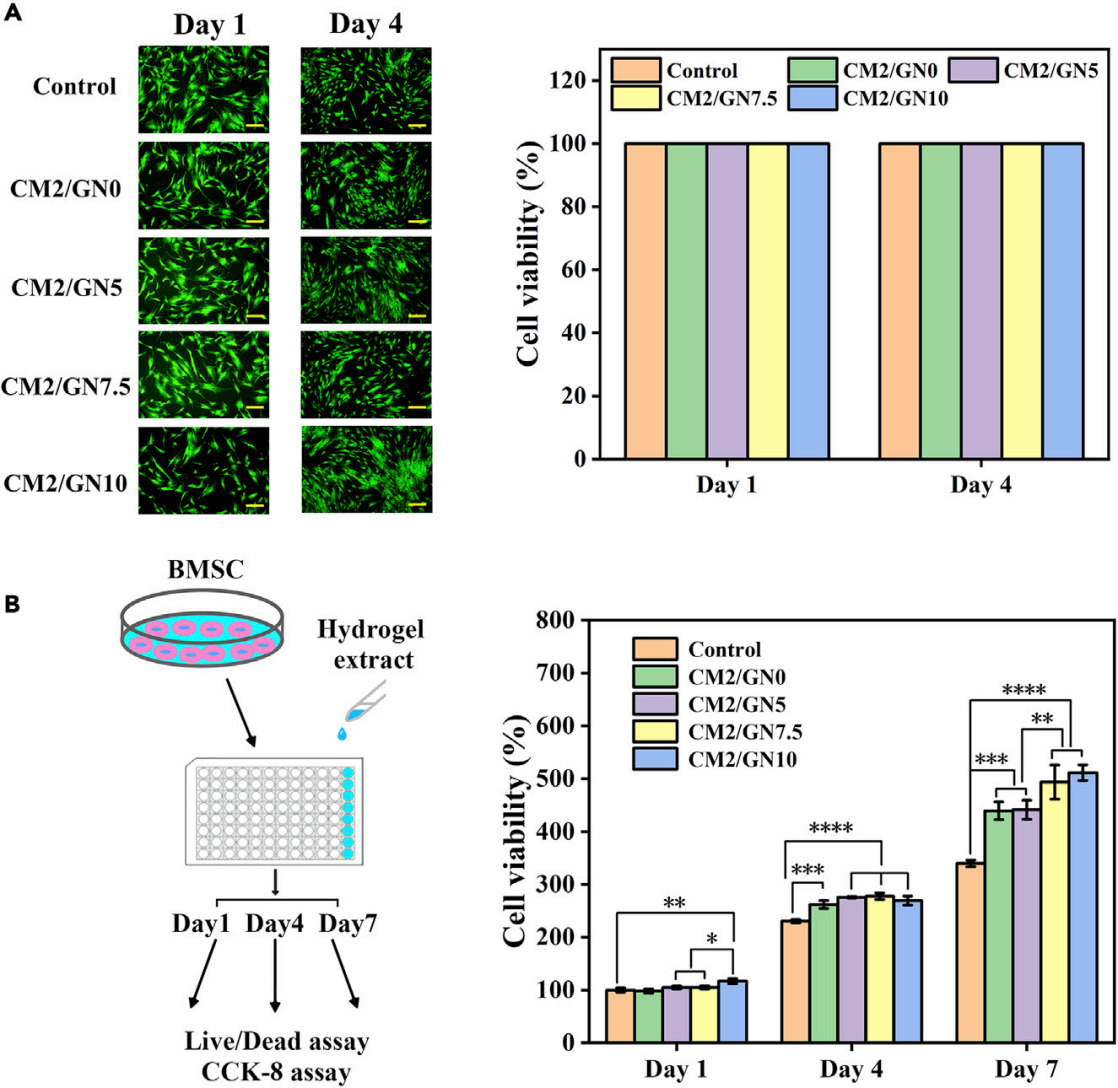
Cytotoxicity of CM/GN hydrogels (A) Live/Dead assay of BMSCs cultured with extract of CM/GN hydrogels at day 1 and day 4: images (left, scale bar: 200 μm) and percentage of viability for each group (right). (B) Proliferation of BMSCs cultured with extract of CM/GN hydrogels. All data are presented as the mean ± SD.

As a species of eligible medical wound dressing, excellent biocompatibility is crucial for clinical application. The experiment of BMSCs Live/Dead assay was conducted to evaluate the cytotoxicity of prepared hydrogels. The green spots represented live BMSCs and red spots represented dead cells (Figure 5A). The viability of cells cultured with CM/GN groups was comparable to that of the control group. These results revealed that the designed CM/GN hydrogels possessed fantastic biocompatibility and non-cytotoxicity to cells, ensuring the prospect to further research for practical adhibition in wound cure. Moreover, the effect that CM/GN contributed to BMSCs growth was studied and the result was shown in Figure 5B. BMSCs proliferation had almost no change between control and CM/GN groups on first day. Obviously, CM/GN facilitated cell proliferation on 4th and 7th day and the promoting effect hoisted with GelNB content increasing. It was explained that gelatin, the raw material for GelNB synthesis, was a species of protein and it would release nutrient substance for cell growth during degradation, leading to the acceleration of BMSCs proliferation (Rogers et al., 2021).

**Figure 6:**
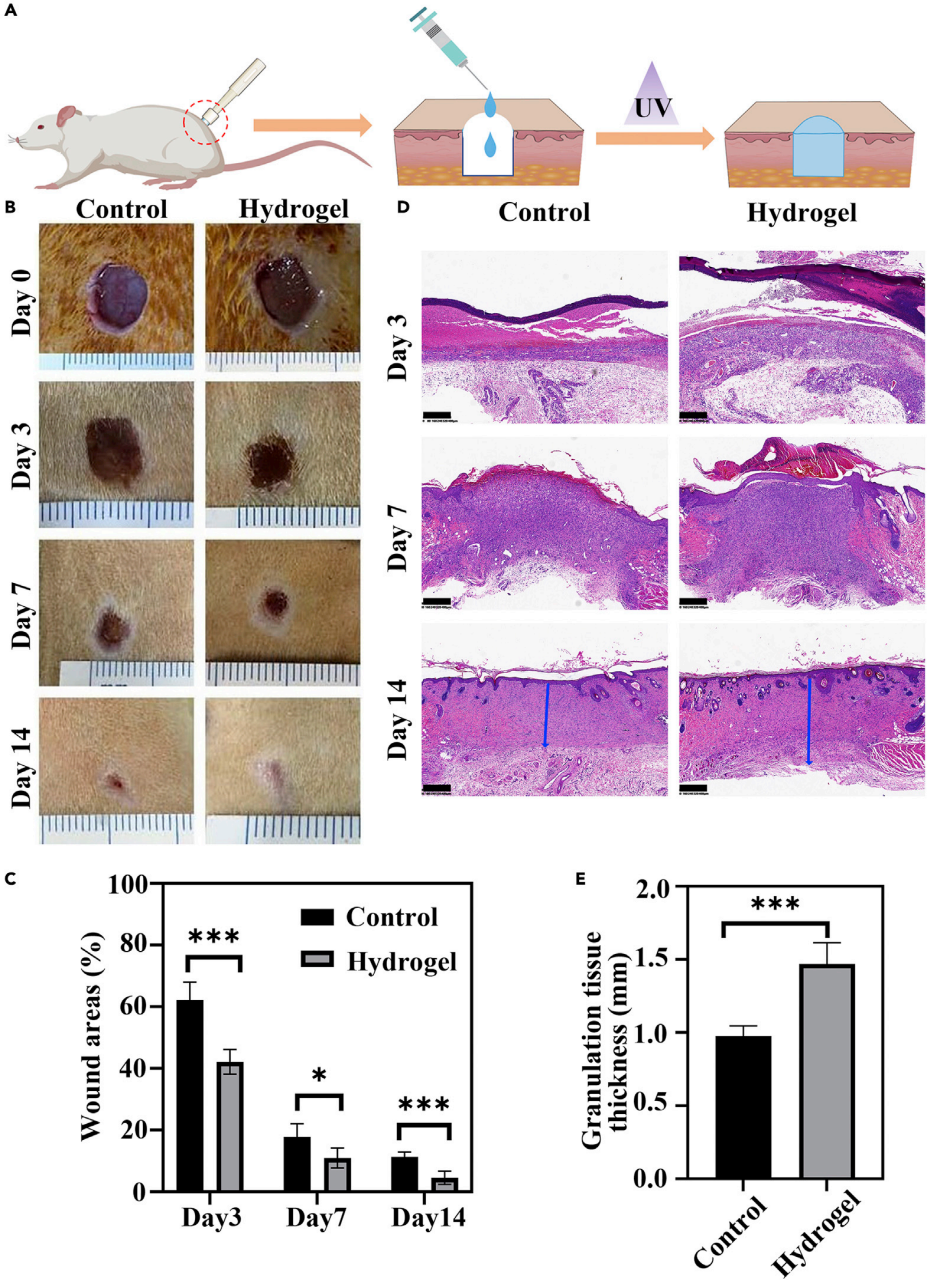
*In vivo* wound healing treated by CM/GN hydrogels (A) Schematic of full-thickness skin defects repair using CM/GN10 hydrogel. (B) Photographs of wounds on 3rd, 7th, and 14th day for hydrogel and control group. (C) Quantitative analysis of the wound areas percentage in all groups (n = 5, ∗p < 0.05, ∗∗∗p < 0.001). (D) Histological evaluation of wound regeneration for hydrogel and control groups on 3rd, 7th, and 14th day. Granulation tissue thickness for hydrogel and control group on 14th day (granulation tissue: blue arrows). Scale bar: 400 μm. (E) Granulation tissue thickness of hydrogel and control groups on 14th day. (n = 5, ∗∗∗p < 0.001). All data are presented as the mean ± SD.

The wound healing performance of the hydrogel wound dressing was further investigated *in vivo* (Figure 6A). Full-thickness skin defects were created on the back of rats. CM/GN10 hydrogel was used to treat the defect, while the control group was treated with PBS. The wound area for hydrogel group and control group were observed on 3rd, 7th, and 14th day and the images were displayed in Figure 6B. On the 3rd and 7th day, both hydrogel group and control group showed reduced wound area, while the hydrogel group exhibited the smaller wound area that demonstrated a comparatively higher wound healing promotion effect. On 14th day, the wound remaining area of both groups was tiny, but hydrogel group was about 62.2% smaller than the control group in wound area. Therefore, our research results show that by tracking the wound area, the wound healing effect of the hydrogel group is better than that of the control group. This could be attributed to the inherent low immunogenicity, antioxidant, antimicrobial, and anti-inflammatory properties of chitosan (Dragostin et al., 2016; Moeini et al., 2020). At the same time, chitosan could affect the hemostasis phase by clotting blood. In this study, in order to reduce the drawbacks of chitosan such as low water solubility and weak mechanical properties, we synthesized MA-functionalized high molecular mass chitosan to overcoming these disadvantages and combined NB-grafted gelatin to fabricate a new biocompatible, photo-crosslinkable, and tissue adhesive hybrid hydrogel.

Wound healing is a well-known biological process comprised of hemostasis, inflammation, proliferation, and remodeling (Moeini et al., 2020). To better understand the wound healing process of CM/GN hydrogel treatment, H&E staining was performed to the tissue sections. As shown in Figure 6D, acute inflammatory responses were observed both for hydrogel groups and control group on the 3rd day, corresponding to the inflammation phase of healing process. Fibroblasts and inflammatory cells migrate to the wound area. There are new blood vessels found in the wound site. Compared with control group, less inflammatory cells and more fibroblast cells were observed around the wound site in the hydrogel-treated group. This result could be attributed to the anti-inflammation of chitosan (Dragostin et al., 2016), and suggesting CM/GN hydrogels promoted the healing process. On the 7th day, a layer of epithelium was found in the hydrogel group. Moreover, higher regularity of connective tissue with fibroblasts was observed in the hydrogel group when compared to control group. When it comes to the 14th day, the stained tissue sections of hydrogel-treated group showed more hair follicles and blood vessels than that of control group. Besides, granulation tissue, containing abundant fibroblasts and growth factors, is another important indicator to assess the wound healing process. As exhibited in Figure 6E, the granulation tissue in hydrogel-treated group was nearly 494 μm thicker than that of control group 14 days post-surgery, demonstrating a superior wound healing effect of CM/GN hydrogel. Taken together, the above results suggested the CM/GN hydrogel promoted wound healing in the rat full-thickness skin defects model.

**Figure 7:**
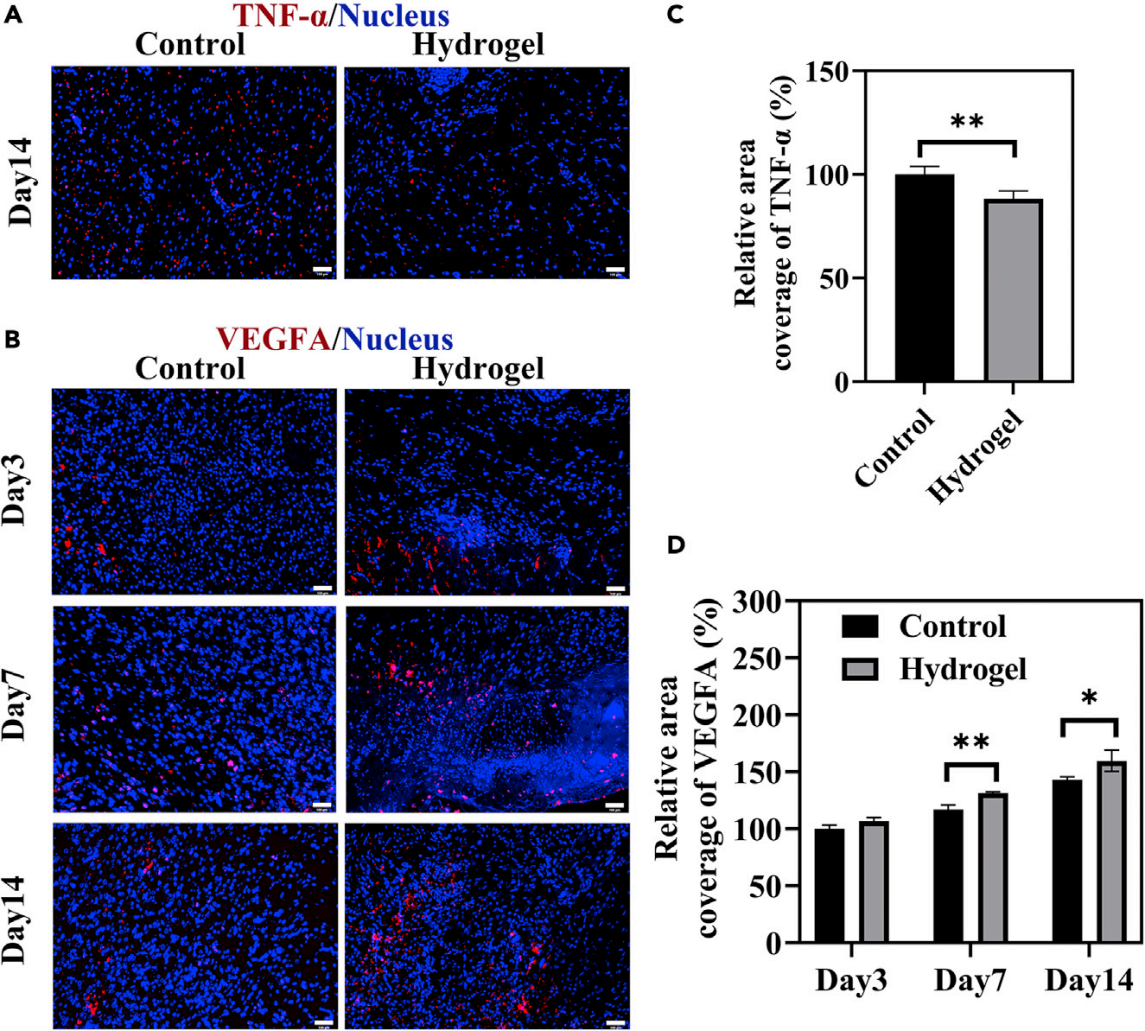
IF staining for the regenerated skin tissues (A) Representative images of skin tissue sections on the 14th day after IF staining labeling with TNF-α (red dots). Scale bar: 100 μm. (B) Representative images of skin tissue sections on the 3rd, 7th, and 14th day after IF staining labeling with VEGF (red dots). Scale bar: 100 μm. (C and D) Quantified analysis of TNF-α (C) and VEGF (D) by calculating the relative percentage of area coverage. ∗p < 0.05, ∗*∗*p < 0.01. All data are presented as the mean ± SD.

Under normal conditions, moderate tumor necrosis factor (TNF) could provide a favorable effect on skin regeneration by enhancing the recruitment of immune cells and promoting the proliferation phase of healing process (Ashcroft et al., 2012). Balanced TNF production is also important for the protective functions when the cutaneous wound is infected. In this study, tumor necrosis factor-α (TNF-α), a typical pro-inflammatory factor, was investigated to evaluate the effect of CM/GN hydrogel in preventing infection. As shown in Figures 7A–7C, significantly higher expression of TNF-α (red dots) was found in the control group than that of the hydrogel-treated group on the 14th day (∗p < 0.05). This could be result from antibacterial property of CM/GN hydrogels. On the other hand, the expression of VEGF was studied because it could regulate collagen synthesis, angiogenesis, and re-epithelization in the wound healing process. Figures 7B–7D shows that, during the regeneration period, higher level of VEGFA (red dots) expression was observed in the hydrogel group when compared with the control group (∗*∗*p < 0.01). Overall, these results indicate the CM/GN hydrogels could accelerate the wound healing process by simultaneously downregulating the production of TNF-α and enhancing VEGF expression.

### Limitations of the study

In this study, we developed a photo-triggering double cross-linked adhesive, antibacterial, and biocompatible composite hydrogel (CM/GN) for promoting full-thickness skin defect repair. CM/GN exhibited a GelNB content-dependent mechanical property and tissue adhesive capacity to porcine skin *ex vivo*. *In vitro* studies based on BMSCs indicated the material was no cytotoxicity and could promote the proliferation of stem cells. Compared with skin healing without treatment, CM/GN could accelerate the healing process and get a fantastic therapeutic effect. Especially, the detection of VEGF and TNF-α proved efficacy of CM/GN in preventing infection and tissue regeneration. Nevertheless, to further demonstrate the antibacterial property of this material, other bacterial species such as *Staphylococcus aureus* could be studied based on the CM/GN hydrogel. Also, additional experiments could be used to reveal the mechanism of the tissue regeneration promoted by biomaterial in gene level. Nonetheless, these results demonstrated that the CM/GN hydrogel was qualified in the field of skin tissue engineering and exhibited the promoting prospect in clinic.

## STAR★Methods

### Key resources table

**Table:** 

REAGENT or RESOURCE	SOURCE	IDENTIFIER
**Chemicals, peptides and recombinant proteins**		

Chitosan (CS)	Qingdao Hecreat Bio-tech company Ltd	CAS: 9012-76-4
Gelatin	Sigma-Aldrich	CAS: 9000-70-8
1-(3-Dimethylaminopropyl)–3-ethylcarbodimide hydrochloride (EDC)	Aladdin (Shanghai, China)	CAS: 25,952-53-8
N-Hydroxysuccinimide (NHS)	Aladdin (Shanghai, China)	CAS:6066-82-6
Sodium bicarbonate (NaHCO_3_)	Aladdin (Shanghai, China)	CAS: 144-55-8
Sodium hydroxide (NaOH)	Aladdin (Shanghai, China)	CAS: 1310-73-2
Methacrylic anhydride (MA)	Sigma-Aldrich	CAS: 760-93-0
Lithium phenyl-2,4,6-trimethylbenzoylphosphinate (LAP)	Haining Jurassic Bio-tech company Ltd	CAS: 85,073-19-4
N-(2-aminoethyl)-4-(4-hydroxymethyl)-2-methoxy-5-nitrosophenoxy) butanamide (NB)	Haining Jurassic Bio-tech company Ltd	
Cell counting Kit-8 (CCK-8)	Beyotime (Shanghai, China)	Cat# C0039
Anti-VEGFA antibody	Proteintech (Wuhang, China)	Cat# 66828-1-Ig; RRID:AB_2882171
Anti-TNFA Ab	Proteintech (Wuhang, China)	Cat# 60291-1-Ig; RRID:AB_2833255
CoraLite488-conjugated Affinipure Goat Anti-Mouse IgG(H + L)	Proteintech (Wuhang, China)	Cat# SA00013-1; RRID:AB_2810983
CoraLite594-conjugated Goat Anti-Rabbit IgG(H + L)	Proteintech (Wuhang, China)	Cat# SA00013-4; RRID:AB_2810984
Sprague Dawley (SD) rats, 2-week-old	Hangzhou Qizhen Laboratory Animal Technology Co., Ltd.(Hangzhou, China)	
DMEM/F12 medium	Keygen Biotech (Jiangsu, China)	Cat# KGM12500N
10% fetal bovine serum	Sigma (USA)	CAS: 1943609-65-1
Penicillin-streptomycin mixture	Keygen Biotech (Jiangsu, China)	Cat# KGY0023
Adult male Sprague Dawley rat	Shanghai SLAC Laboratory Animal Co., Ltd. (Shanghai, China)	

### Resource availability

#### Lead contact

Further information and requests for resources and reagents should be directed to and will be fulfilled by the lead contact, Wei Wei (zjewwei@zju.edu.cn).

#### Materials availability

All materials are from commercial sources and are widely available.

### Experimental model and subject details

#### Cell lines

The 2-week-old male Sprague Dawley (SD) rats were used as donors to culture primary rBMSCs. Rats were anesthetized by inhalation of 3% isoflurane and then euthanized by intraperitoneal injection of sodium pentobarbital (70 mg/kg). The long bones were obtained by removing the skin of the two hind legs and dissecting them at the hip, knee, and ankle joints. Femur and tibia with residual tissue removed were rinsed with phosphate-buffered saline (PBS, Keygen Biotech, China). The prepared femur and tibia were immersed in DMEM/F12 medium (Keygen Biotech, China) with 10% fetal bovine serum (FBS, Sigma, USA) and 1% penicillin-streptomycin mixture (Keygen Biotech, China). Bone marrow was collected by continuous rinsing with DMEM/F12 medium after insertion of a 22-gauge needle into the midsection of the femur and tibia. Collect the bone marrow cell suspension in a sterile 15 mL centrifuge tube and then centrifuge at 1000 rpm for 3 min. Resuspend the pellet culture medium. The suspended cells were cultured in a T25 cm^2^ flask and placed in a 37°C incubator containing 5.0% CO_2_. Replace the medium every 3 days. All experiments used BMSCs at passage 3.

### Method details

#### Synthesis of CSMA

The CSMA was synthesized according to previously reported literature (Kufelt et al., 2015). Briefly, CS was first dissolved in acetic acid to form 1 wt % aqueous solution. MA was then added into the solution (anhydride: amino = 1: 1) at 60°C and stirred for 6 h. Then, the solution was neutralized with 10 wt% NaHCO_3_. The synthesized CSMA was dialyzed against deionized water for 4 days and final product was obtained by lyophilization.

#### Synthesis of GelNB

GelNB was fabricated through the amidate between -COOH of NB and -NH_2_ of gelatin (Zhang et al., 2021). In brief, EDC/NHS and NB were dissolved in dimethyl sulfoxide (DMSO) and the mixture was added into homogeneous gelatin solution slowly. The system was kept at 45°C and stirred for 4 h. The product was purified by deionized water dialysis for 3 days. The synthesized GelNB solution was collected and freeze-dried for the following studies.

#### Fabrication of hydrogels

The process of the photo-triggering hydrogels was displayed in Figure 1. Different amounts of freeze-drying GelNB were dissolved in 2 wt% CMSA solution at 55°C and 0.5 wt% (final concentration) photo-initiator LAP was added. The precursor mixture was transferred into a custom-made polydimethylsiloxane (PDMS) mode (Ф10 mm × 2 mm) and irradiated under UV light for 5–20 s to generate the hydrogels (CM2/GN0, CM2/GN5, CM2/GN7.5, CM2/GN10) (see Table S1).

#### Characterization

^1^H Nuclear Magnetic Resonance (^1^H-NMR) spectra of CSMA and GelNB was obtained by an AV-300 NMR spectrometer (Bruker, 400 MHz). The chemical structures of freeze-dried hydrogels were characterized by Fourier transform infrared (FT-IR) spectroscopy (NICOLET IS10 spectrometer) in the region of 4000 and 400 cm^−1^ (also see Figure S1). The microstructures of hydrogels were observed by ZEISS SIGMA 500 scanning electron microscope (SEM). The rheology tests were performed using a rheometer (Anton Paar MCR302).

#### Mechanical characterization

Compressive tests of CM/GN hydrogels were performed under an electronic testing machine (MTS C41) with a 50 N load cell. The samples were cured in a pre-manufactured polydimethylsiloxane (PDMS) mold (10 mm in diameter, 2 mm in depth). The compressive rate was set as 1 mm/min. The compressive modulus was calculated from the linear region of the stress-strain curve.

#### Lap shear test

The lap shear strength of the CM/GN hydrogels was measured under a modified ASTM standard (F2255-05). The substrates were glass slides covered by collagen sausage casing. 10 μL of CM/GN pre-gel solution was pipetted between two substrates (overlapped area was 25 × 10mm). The adhesive was achieved upon UV exposure. The substrates were loaded to breakage using a testing machine (MTS C41) at a speed of 5 mm/min.

#### Test of water content

The prepared hydrogels were lyophilized and the weights of the hydrogels before drying (W_0_) and after drying (W_d_) were recorded. Water content (W_c_) was defined as the following equation and the results were shown in Figure S2:

(Equation 1) $$W_{c}=\frac{W_{0}-W_{d}}{W_{0}}$$

#### Swelling kinetic studies

A slice of freeze-dried hydrogel (m_0_) was immersed in 10 mL deionized water at room temperature. Then, the hydrogels were taken out and wiped with filter paper at required time. The weights (m_t_) were measured and record until they approached constant. Swelling ratio was calculated as the following equation:

(Equation 2) $$Swelling\;ratio=\frac{m_{t}}{m_{0}}$$

#### Deswelling kinetic studies

Deswelling kinetic experiment was conducted by measuring the weights of the fully swollen hydrogels exposed to air at room temperature at pre-determined time intervals. Deswelling ratio was defined as the following equation:

(Equation 3) $$Deswelling\;ratio=\frac{m_{t}}{m_{e}}$$

Where m_t_ represented the weights of hydrogels measured at time t and m_e_ represented the weights of fully swollen hydrogels.

#### Antibacterial performance

*E. coli* (initial OD value was 0.03) LB culture was spread onto the surface of agarose gel evenly and CM/GN hydrogels were put on the culture medium. The area that *E. coli* was killed represented antibacterial effect and it was calculated as the following equation:

(Equation 4) $$Antibacterial\;effect=\frac{S_{g}}{S_{0}}$$

Where S_g_ represented the area the CM/GN could influence *E. coli* reproduction and S_0_ represented the size of CM/GN.

Moreover, another experiment to evaluate the hydrogels antibacterial capacity was conducted. A piece of prepared hydrogel was immersed in 5 mL *E. coli* LB culture (initial OD value was 0.03) and it was stirred at 37°C. OD values was recorded at required time and antibacterial capacity was exhibited.

#### Cell viability and proliferation

The designed precursor solution was sterilized by 0.22 μm filters and photocured in a sterile environment. The prepared hydrogels were first immersed in Dulbecco’s modified Eagle’s medium (DMEM) for 12 h to make a hydrogel extract. Then, rat BMSCs were seeded in normal cell culture plates, cultured with the extract, and incubated at 37°C and 5% CO_2_. The cells were also treated by a Live/Dead assay and imaged on an inverted fluorescence microscope at pre-determined time points. Meanwhile, the BMSCs were treated by CCK-8 assay according to the instruction to evaluate the cell proliferation. The OD values were measured by a microplate reader.

#### *In vivo* skin defects model

All animals were treated according to the standard guidelines approved by the Zhejiang University Ethics Committee (ZJU20210204). For the evaluation of the wound healing effect of the hydrogel wound dressing, a rat (adult male Sprague Dawley rat, 6–8 weeks, 200–300 g) full-thickness skin defect model was used. All rats were acclimatized for 7 days before surgery. The animals were injected of sodium pentobarbital (40–50 mg/kg body weight) with intraperitoneal injection for anesthesia. Under aseptic condition, full thickness skin round wounds (10 mm in diameter) were created by a needle biopsy on the shaved dorsal region of rats above the tail but below the back. After that, the hydrogel group was injected with hydrogel wound dressing followed by a UV irradiation (30 mW/cm^2^) for 20 s. The control group were treated with 40 μL of PBS and wound was bandaged with a clean dressing. The rats were allowed to move freely in the cages in the following days. Photos of the wound area were recorded every day. On the 3rd, 7th and 14th day after surgery, rats in each group (n = 5) were sacrificed by an overdose of anesthetic, and the wound skin tissue was excised for histological analysis.

#### Histological analysis

To evaluate the inflammation and epidermal regeneration in the wound area, tissues containing the wound site and their surrounding healthy skin were collected. The tissue samples were fixed in 4% (v/v) paraformaldehyde for 1 h right after sacrifice before embedded in paraffin. The samples were cross sectioned to slices (4 μm thickness) and then stained by Hematoxylin-Eosin (H&E). All slides were scanned and analyzed by a Digital Slide Scanner (KFBIO, Ningbo). The regenerated skins from the wound site were also excised for IF staining with Anti-VEGFA antibody (proteintech) and TNFA Ab (proteintech), respectively. CoraLite488-conjugated Affinipure Goat Anti-Mouse IgG(H + L) (proteintech) and CoraLite594-conjugated Goat Anti-Rabbit IgG(H + L) (proteintech) were used as the secondary antibody to reveal VEGFA and TNFA expression. The nuclei were stained with 4′,6-diamidino-2-phenylindole. Slides were observed under an upright fluorescence microscope (BX53, Olympus).

#### Statistical analysis

All data are presented as the mean ± SD. Differences between the values were evaluated using one-way ANOVA or Student’s t-test. ∗p < 0.05, ∗∗p < 0.01, ∗∗∗p < 0.001, ∗∗∗∗p < 0.0001 was considered statistically significant.

## Data Availability

Original data are available from corresponding authors. This paper does not report the original code. Any additional information required to reanalyze the data reported in this paper is available from the lead contact upon request.

## References

[bib1] Ahmad T., Ismail A., Ahmad S.A., Khalil K.A., Kumar Y., Adeyemi K.D., Sazili A.Q. (2017). Recent advances on the role of process variables affecting gelatin yield and characteristics with special reference to enzymatic extraction: a review. Food Hydrocolloids..

[bib2] Ashcroft G.S., Jeong M.J., Ashworth J.J., Hardman M., Jin W., Moutsopoulos N., Wild T., McCartney-Francis N., Sim D., McGrady G. (2012). Tumor necrosis factor-alpha (TNF-alpha) is a therapeutic target for impaired cutaneous wound healing. Wound Repair Regen..

[bib3] Atashgah R.B., Ghasemi A., Raoufi M., Abdollahifar M.A., Zanganeh S., Nejadnik H., Abdollahi A., Sharifi S., Lea B., Cuerva M. (2021). Restoring endogenous repair mechanisms to heal chronic wounds with a multifunctional wound dressing. Mol. Pharm..

[bib4] Attasgah R.B., Velasco-Rodríguez B., Pardo A., Fernández-Vega J., Arellano-Galindo L., Rosales-Rivera L.C., Prieto G., Barbosa S., Soltero J.F.A., Mahmoudi M., Taboada P. (2022). Development of functional hybrid scaffolds for wound healing applications. iScience.

[bib5] Blacklow S.O., Li J., Freedman B.R., Zeidi M., Mooney D.J., Mooney D.J. (2019). Bioinspired mechanically active adhesive dressings to accelerate wound closure. Sci. Adv..

[bib6] Balakrishnan B., Joshi N., Jayakrishnan A., Banerjee R. (2014). Self-crosslinked oxidized alginate/gelatin hydrogel as injectable, adhesive biomimetic scaffolds for cartilage regeneration. Acta Biomater..

[bib7] Choi J.R., Lee J.H., Xu A., Matthews K., Xie S., Duffy S.P., Ma H. (2020). Monolithic hydrogel nanowells-in-microwells enabling simultaneous single cell secretion and phenotype analysis. Lab Chip.

[bib8] Douglas K.L., Piccirillo C.A., Tabrizian M. (2006). Effects of alginate inclusion on the vector properties of chitosan-based nanoparticles. J. Control. Release.

[bib9] Dragostin O.M., Samal S.K., Dash M., Lupascu F., Pânzariu A., Tuchilus C., Ghetu N., Danciu M., Dubruel P., Pieptu D. (2016). New antimicrobial chitosan derivatives for wound dressing applications. Carbohydr. Polym..

[bib10] Duan Y., He K., Zhang G., Hu J. (2021). Photoresponsive micelles enabling codelivery of nitric oxide and formaldehyde for combinatorial antibacterial applications. Biomacromolecules.

[bib11] Fairbanks B.D., Schwartz M.P., Bowman C.N., Anseth K.S. (2009). Photoinitiated polymerization of PEG-diacrylate with lithium phenyl-2, 4, 6-trimethylbenzoylphosphinate: polymerization rate and cytocompatibility. Biomaterials.

[bib12] Fan Z., Liu B., Wang J., Zhang S., Lin Q., Gong P., Ma L., Yang S. (2014). A novel wound dressing based on Ag/Graphene polymer hydrogel: effectively kill bacteria and accelerate wound healing. Adv. Funct. Mater..

[bib13] Geng X., Kwon O.H., Jang J. (2005). Electrospinning of chitosan dissolved in concentrated acetic acid solution. Biomaterials.

[bib14] Ghorbani M., Roshangar L., Soleimani Rad J. (2020). Development of reinforced chitosan/pectin scaffold by using the cellulose nanocrystals as nanofillers: an injectable hydrogel for tissue engineering. Eur. Polym. J..

[bib15] Gong C., Wu Q., Wang Y., Zhang D., Luo F., Zhao X., Wei Y., Qian Z. (2013). A biodegradable hydrogel system containing curcumin encapsulated in micelles for cutaneous wound healing. Biomaterials.

[bib16] Guo B., Glavas L., Albertsson A.-C. (2013). Biodegradable and electrically conducting polymers for biomedical applications. Prog. Polym. Sci..

[bib17] Heo D.N., Alioglu M.A., Wu Y., Ozbolat V., Ayan B., Dey M., Kang Y., Ozbolat I.T. (2020). 3D Bioprinting of carbohydrazide-modified gelatin into microparticle-suspended oxidized alginate for the fabrication of complex-shaped tissue constructs. ACS Appl. Mater. Interfaces.

[bib18] Honda Y., Takeda Y., Li P., Huang A., Sasayama S., Hara E., Uemura N., Ueda M., Hashimoto M., Arita K. (2018). Epigallocatechin gallate-modified gelatin sponges treated by vacuum heating as a novel scaffold for bone tissue engineering. Molecules.

[bib19] Hong Y., Zhou F., Hua Y., Zhang X., Ni C., Pan D., Zhang Y., Jiang D., Yang L., Lin Q. (2019). A strongly adhesive hemostatic hydrogel for the repair of arterial and heart bleeds. Nat. Commun..

[bib20] Huang T., Tu Z.-c., Shangguan X., Sha X., Wang H., Zhang L., Bansal N. (2019). Fish gelatin modifications: a comprehensive review. Trends Food Sci. Technol..

[bib21] Karnnet S., Potiyaraj P., Pimpan V. (2005). Preparation and properties of biodegradable stearic acid-modified gelatin films. Polym. Degrad. Stab..

[bib22] Koivusalo L., Kauppila M., Samanta S., Parihar V.S., Ilmarinen T., Miettinen S., Oommen O.P., Skottman H. (2019). Tissue adhesive hyaluronic acid hydrogels for sutureless stem cell delivery and regeneration of corneal epithelium and stroma. Biomaterials.

[bib23] Kolawole O.M., Lau W.M., Khutoryanskiy V.V. (2018). Methacrylated chitosan as a polymer with enhanced mucoadhesive properties for transmucosal drug delivery. Int. J. Pharm..

[bib24] Kufelt O., El-Tamer A., Sehring C., Meißner M., Schlie-Wolter S., Chichkov B.N. (2015). Water-soluble photopolymerizable chitosan hydrogels for biofabrication via two-photon polymerization. Acta Biomater..

[bib25] Kwak K.-S., Cho S.-M., Ji C.-I., Lee Y.-B., Kim S.-B. (2009). Changes in functional properties of shark (Isurus oxyrinchus) cartilage gelatin produced by different drying methods. Int. J. Food Sci. Technol..

[bib26] Liu H., Li Z., Zhao Y., Feng Y., Zvyagin A.V., Wang J., Yang X., Yang B., Lin Q. (2021). Novel diabetic foot wound dressing based on multifunctional hydrogels with extensive temperature-tolerant, durable, adhesive, and intrinsic antibacterial properties. ACS Appl. Mater. Interfaces.

[bib27] Mao X., Mao D., Jiang J., Su B., Chen G., Zhu X. (2021). A semi-dry chemistry hydrogel-based smart biosensing platform for on-site detection of metal ions. Lab Chip.

[bib28] Matoori S., Veves A., Mooney D.J. (2021). Advanced bandages for diabetic wound healing. Sci. Transl. Med..

[bib29] Mitropoulos A.N., Marelli B., Ghezzi C.E., Applegate M.B., Partlow B.P., Kaplan D.L., Omenetto F.G. (2015). Transparent, nanostructured silk fibroin hydrogels with tunable mechanical properties. ACS Biomater. Sci. Eng..

[bib30] Moeini A., Pedram P., Makvandi P., Malinconico M., Gomez d'Ayala G. (2020). Wound healing and antimicrobial effect of active secondary metabolites in chitosan-based wound dressings: a review. Carbohydr. Polym..

[bib31] Nguyen M.K., Alsberg E. (2014). Bioactive factor delivery strategies from engineered polymer hydrogels for therapeutic medicine. Prog. Polym. Sci..

[bib32] Niu G., Choi J.S., Wang Z., Skardal A., Giegengack M., Soker S. (2014). Heparin-modified gelatin scaffolds for human corneal endothelial cell transplantation. Biomaterials.

[bib33] Osi A.R., Zhang H., Chen J., Zhou Y., Wang R., Fu J., Müller-Buschbaum P., Zhong Q. (2021). Three-dimensional-printable thermo/photo-cross-linked methacrylated chitosan-gelatin hydrogel composites for tissue engineering. ACS Appl. Mater. Interfaces.

[bib34] Qi X., Hu X., Wei W., Yu H., Li J., Zhang J., Dong W. (2015). Investigation of Salecan/poly (vinyl alcohol) hydrogels prepared by freeze/thaw method. Carbohydr. Polym..

[bib35] Rogers R.E., Haskell A., White B.P., Dalal S., Lopez M., Tahan D., Pan S., Kaur G., Kim H., Barreda H. (2021). A scalable system for generation of mesenchymal stem cells derived from induced pluripotent cells employing bioreactors and degradable microcarriers. Stem Cells Transl. Med..

[bib36] Rose J.B., Pacelli S., Haj A.J.E., Dua H.S., Hopkinson A., White L.J., Rose F. (2014). Gelatin-based materials in ocular tissue engineering. Materials.

[bib37] Suderman N., Isa M.I.N., Sarbon N.M. (2018). The effect of plasticizers on the functional properties of biodegradable gelatin-based film: a review. Food Biosci..

[bib38] Sun X., Dong M., Guo Z., Zhang H., Wang J., Jia P., Bu T., Liu Y., Li L., Wang L. (2021). Multifunctional chitosan-copper-gallic acid based antibacterial nanocomposite wound dressing. Int. J. Biol. Macromol..

[bib39] Van Nieuwenhove I., Salamon A., Peters K., Graulus G.J., Martins J.C., Frankel D., Kersemans K., De Vos F., Van Vlierberghe S., Dubruel P. (2016). Gelatin- and starch-based hydrogels. Part A: hydrogel development, characterization and coating. Carbohydr. Polym..

[bib40] Wei W., Hu X., Qi X., Yu H., Liu Y., Li J., Zhang J., Dong W. (2015). A novel thermo-responsive hydrogel based on salecan and poly (N-isopropylacrylamide): synthesis and characterization. Colloids Surf. B Biointerfaces.

[bib41] Wei W., Ma Y., Zhang X., Zhou W., Wu H., Zhang J., Lin J., Tang C., Liao Y., Li C. (2021). Biomimetic joint paint for efficient cartilage repair by simultaneously regulating cartilage degeneration and regeneration in pigs. ACS Appl. Mater. Interfaces.

[bib42] Xavier J.R., Thakur T., Desai P., Jaiswal M.K., Sears N., Cosgriff-Hernandez E., Kaunas R., Gaharwar A.K. (2015). Bioactive nanoengineered hydrogels for bone tissue engineering: a growth-factor-free approach. ACS Nano.

[bib43] Xu R., Luo G., Xia H., He W., Zhao J., Liu B., Tan J., Zhou J., Liu D., Wang Y. (2015). Novel bilayer wound dressing composed of silicone rubber with particular micropores enhanced wound re-epithelialization and contraction. Biomaterials.

[bib44] Yang Y., Zhang J., Liu Z., Lin Q., Liu X., Bao C., Wang Y., Zhu L. (2016). Tissue-integratable and biocompatible photogelation by the imine crosslinking reaction. Adv. Mater..

[bib45] Yang Z., Huang R., Zheng B., Guo W., Li C., He W., Wei Y., Du Y., Wang H., Wu D., Wang H. (2021). Highly stretchable, adhesive, biocompatible, and antibacterial hydrogel dressings for wound healing. Adv. Sci..

[bib46] Yin J., Yan M., Wang Y., Fu J., Suo H. (2018). 3D bioprinting of low-concentration cell-laden gelatin methacrylate (GelMA) bioinks with a two-step cross-linking strategy. ACS Appl. Mater. Interfaces.

[bib47] Yue K., Trujillo-de Santiago G., Alvarez M.M., Tamayol A., Annabi N., Khademhosseini A. (2015). Synthesis, properties, and biomedical applications of gelatin methacryloyl (GelMA) hydrogels. Biomaterials.

[bib48] Yuk H., Zhang T., Lin S., Parada G.A., Zhao X. (2016). Tough bonding of hydrogels to diverse non-porous surfaces. Nat. Mater..

[bib49] Zhang Y., Li C., Zhu Q., Liang R., Xie C., Zhang S., Hong Y., Ouyang H. (2021). A long-term retaining molecular coating for corneal regeneration. Bioact. Mater..

[bib50] Zhao X., Wu H., Guo B., Dong R., Qiu Y., Ma P.X. (2017). Antibacterial anti-oxidant electroactive injectable hydrogel as self-healing wound dressing with hemostasis and adhesiveness for cutaneous wound healing. Biomaterials.

[bib51] Zhao Z., Wang Z., Li G., Cai Z., Wu J., Wang L., Deng L., Cai M., Cui W. (2021). Injectable microfluidic hydrogel microspheres for cell and drug delivery. Adv. Funct. Mater..

