# Supplementary material for: A photo-triggering double cross-linked adhesive, antibacterial, and biocompatible hydrogel for wound healing
Source: iScience. 2022 Jun 16;25(7):104619. doi: 10.1016/j.isci.2022.104619 (PMC9250026; doi:10.1016/j.isci.2022.104619)
Supplement: Document S1. Figures S1 and S2 and Table S1 [file mmc1.pdf]

iScience, Volume *25*

Supplemental information

A photo-triggering double cross-linked adhesive, antibacterial, and biocompatible hydrogel for wound healing

Honghua Hu, Xinrang Zhai, Wenyue Li, Shunxian Ji, Wei Dong, Weiyu Chen, Wei Wei, and Zhongfa Lu

Supplemental Information

Figures

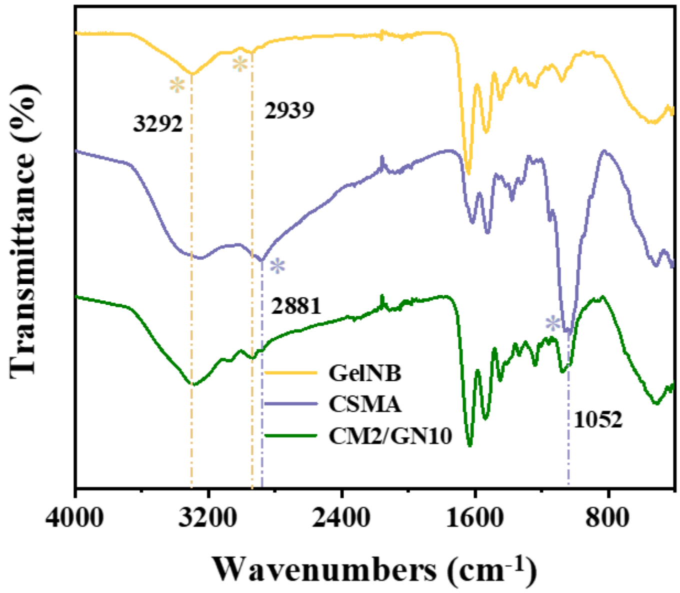

Fig. S1. Characterizations of hydrogels. FT-IR spectra of prepared GelNB, CSMA, and CM2/GN10 hydrogel.

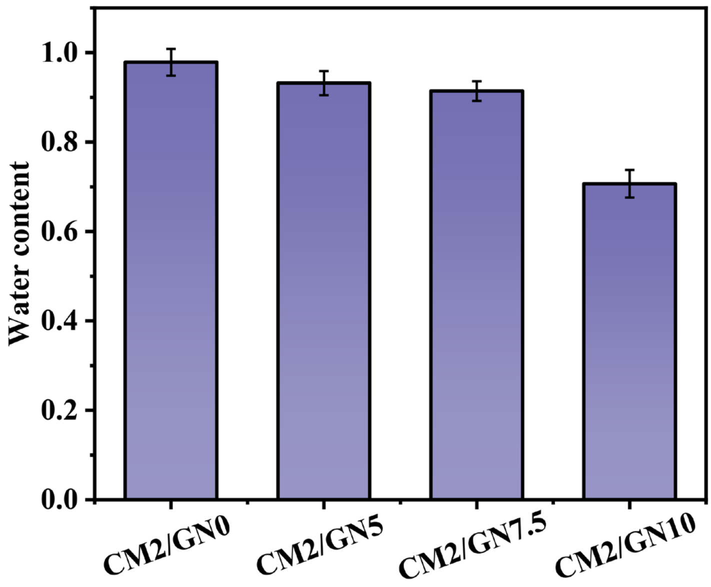

Fig. S2. Swelling properties of hydrogels. Water content of different CM/GN hydrogels.

Table. S1. **Composition ratios used to fabricate the CM/GN hydrogels**, Related to STAR Methods

**Sample Codes**	**CSMA (CM)** ***(wt/vol)***	**GelNB (GN)** ***(wt/vol)***	**Gelation Time (37°C)**
**CM2/GN0**	**2%**	**0**	**～15s**
**CM2/GN5**	**2%**	**5%**	**～10s**
**CM2/GN7.5**	**2%**	**7.5%**	**～5s**
**CM2/GN10**	**2%**	**10%**	**～5s**
